# Unexpected intraoperative finding of gallbladder torsion

**DOI:** 10.1093/jscr/rjab478

**Published:** 2021-12-11

**Authors:** P Sai Krishna, R D R Somasekar, A Siva Sankar

**Affiliations:** Department of Surgical Gastroenterology, GMKMCH, Salem 636002, India; Department of Surgical Gastroenterology, GMKMCH, Salem 636002, India; Department of Surgical Gastroenterology, GMKMCH, Salem 636002, India

## Abstract

Gallbladder (GB) torsion or volvulus is a rare entity affecting elderly women. Only ~500 cases have been reported in the literature. Incidence is rare seen in ~1 in 365 520.

A constant finding is the presence of the GB on a mobile mesentery. Torsion, or volvulus, of the GB occurs when it twists axially, with the subsequent obstruction of bile and/or blood flow.

We briefly describe a 75-year-old female patient with acute abdomen and ultrasound and Computed tomography of abdomen revealed a distended GB. On laparotomy, we encountered a twisted GB with gangrene and cholecystectomy was done.

GB volvulus is a rare occurrence and clinically mimic’s acute cholecystitis and should be sought with high suspicion especially when encountering a thin elderly woman. Immediate diagnosis is prime as delay may be fatal.

Even with recent advances in imaging, it is difficult to make a correct preoperative diagnosis of GB torsion.

## INTRODUCTION

Gallbladder (GB) torsion is a very rare clinical entity and was first described in 1896 by Wendel [1]. It is commonly seen in elderly women with thin body type [2, 3]. In literature only 500 cases reported and the recent increase in cases may be because of increased mean lifetime [4].

The exact etiology of GB torsion remains unknown, although certain anatomical variants predisposing to this condition are well established [5]. First, the GB has its own mesentery and second, the cystic duct and artery have a mesentery, and the GB is free within the peritoneal cavity. These congenital variants are thought to be exacerbated following liver atrophy and loss of visceral fat [6].

Torsion can be of two types. Incomplete, in which rotation is ≤180°, or complete, in which rotation is >180°. Both clockwise and anticlockwise rotations have been described; it has been proposed that clockwise rotation occurs as a result of gastric and duodenal peristalsis, whereas anticlockwise rotation is secondary to colonic peristalsis [7].

This clinical entity is indistinguishable from those of acute cholecystitis. There have been a limited number of case reports in which a preoperative diagnosis of GB torsion was made promptly.

We present a case of torsion of GB torsion which was equivocal on imaging and was confirmed by laparotomy and cholecystectomy was performed.

## CASE PRESENTATION

A 75-year-old female patient was admitted to emergency room with complaints of acute abdominal pain, nausea for 2 days.

On physical examination, tenderness and guarding was found in umbilical region. Murphy’s sign was negative. On complete blood examination, WBC count was 14 400/mm^3^ and other biochemical parameters were normal.

**Figure 1:**
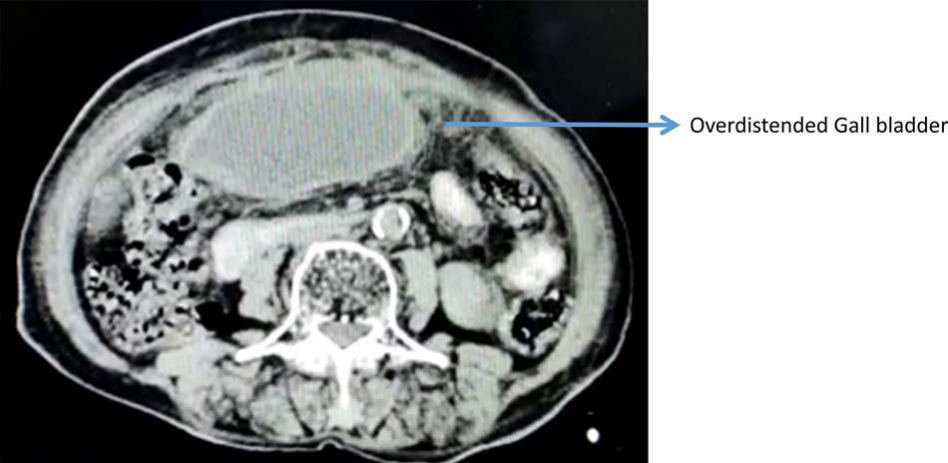
Ultrasonography of abdomen showing over distended GB.

Over distended gall bladder with thickened laminated walls filled with thick internal echoes suggestive of acute cholecystitis without gall stones were the main findings of abdominal ultrasonography (Fig. 1). Color Doppler showed no intramural blood flow in the wall of the GB.

**Figure 2:**
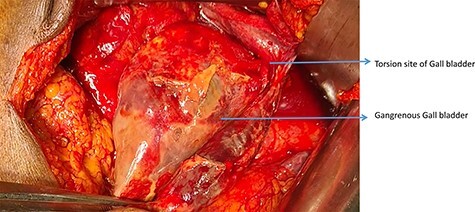
Contrast enhanced computed tomography abdomen showing over distended GB.

On computerized tomography, there was no additional finding (Fig. 2).

Subsequently an abdominal exploration decision was made under the guidance of physical examination signs. A median laparotomy was performed after obtaining a written informed consent from the patient.

Laparoscopy for acute cholecystitis showed a reduced overall morbidity, reduced wound complications and pneumonia with reduced length of hospital stay [9].

However, laparoscopy was not available in emergency setting in our care center, and hence a median laparotomy was performed after obtaining a written informed consent from the patient.

**Figure 3:**
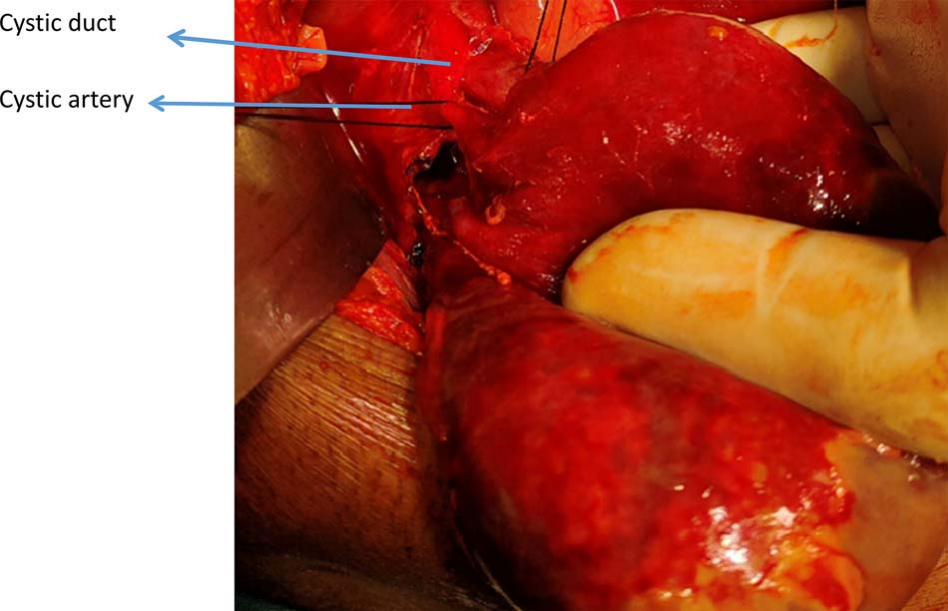
Intraoperative picture showing Torsion site of GB.

**Figure 4:**
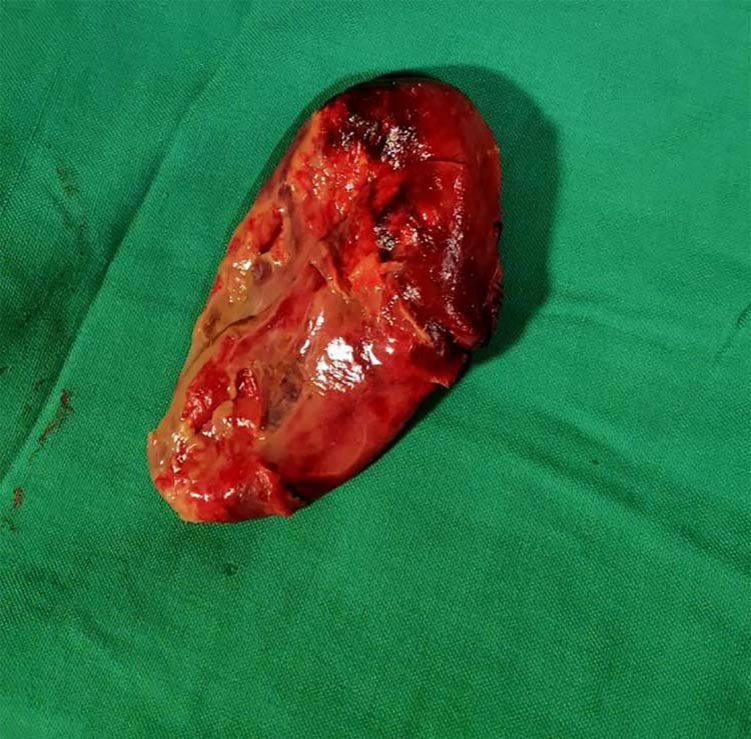
Derotated GB showing Cystic duct and artery.

There was purulent fluid collection in the right upper quadrant. GB was gangrenous and congested. GB had a loose mesentery and was torsioned by a rotation of 180° clockwise (Figs 3 and 4). Cholecystectomy was performed after the detorsion of GB. There was no additional finding.

**Figure 5:**
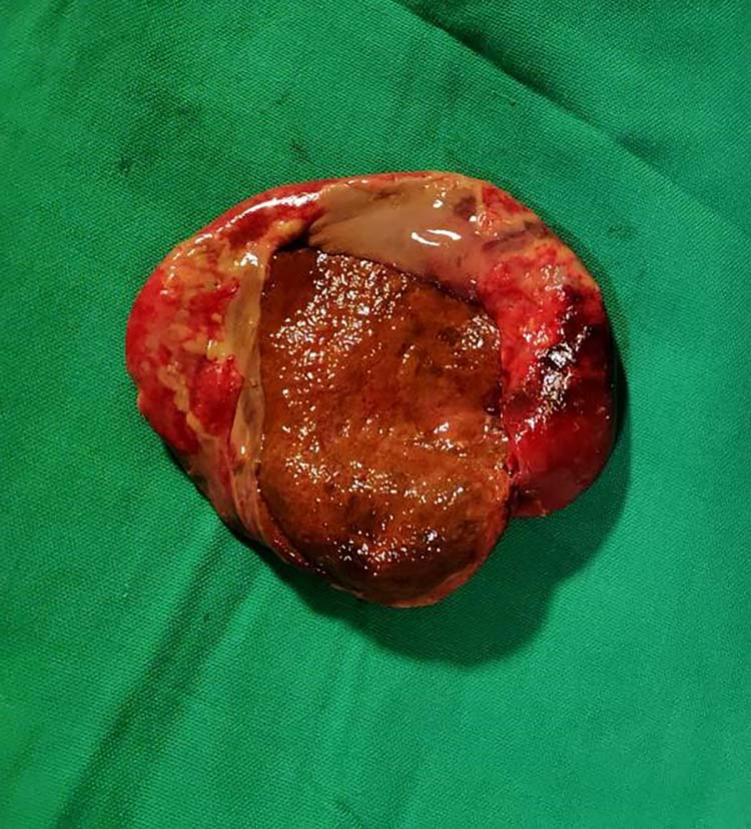
Gross and cut open showing gangrenous mucosa.

Cut open of the specimen shows gangrenous mucosa (Fig. 5).

Patient postoperative period was uneventful and discharged on pod 4.

## DISCUSSION

Torsion of GB is an uncommon occurrence with high predilection to elderly with female to male ratio of 3:1 [8].

The etiology of GB volvulus is unknown but some underlying factors seem to be present. First the GB is usually ‘floating’ that is where the entire organ is in contact with the peritoneum and it is connected to the porta hepatis only by the cystic pedicle. A congenital anomaly may be present where an abnormally long mesentery can suspend the GB away from the liver bed, thus increasing the chance for torsion. Likewise relaxation and atrophy of previously normal mesentery can cause visceroptosis.

But for the torsion to occur a triggering event has to occur. Liver atrophy, loss of visceral fat and elasticity, weight loss, atherosclerosis of the cystic artery and spinal deformities have all been theorized as possible triggering factors [3, 8]. Interesting to note that our patient was very thin weighing just under 55 kilograms.

Symptoms of GB torsion are usually nonspecific and mimic those of acute cholecystitis. There may be complete or incomplete torsion. Torsion with a degree of rotation of <180 is defined as incomplete and it mimics biliary colic.

In complete torsion, GB perfusion is deteriorated, and patients show the signs of acute cholecystitis. Examination findings may be nonspecific, but many will have clinical signs of acute cholecystitis at admission. Laboratory studies are usually nondiagnostic.

Preoperative diagnosis is difficult with fewer than a dozen cases found in the literature. Most diagnoses are made intraoperatively.

In this case, we performed a laparotomy due to the equivocal nature of the imaging studies and we found a long mesentery with 180° clockwise torsion with gangrenous gall bladder and cholecystectomy was performed.

## CONCLUSION

GB volvulus is a rare occurrence should be suspected in all patients presenting with signs and symptoms of acute cholecystitis, especially if they are elderly thin women. Delay in treatment can lead to necrosis, perforation and peritonitis and hence immediate diagnosis and management are of prime importance.

## AUTHOR’S INFORMATION

Study concept and design—Dr P. Sai Krishna

Data collection—Dr P. Sai Krishna

Editing—Dr R.D.R Somasekar

Data analysis or interpretation—Dr A. Sivasankar

Writing the paper—Dr R.D.R Somasekar

## CONFLICT OF INTEREST STATEMENT

None declared.

## FUNDING

None.

## ETHICAL APPROVAL

Ethical approval was obtained—Committee—Govt. Mohan Kumaramangalam Medical College Hospital.

## CONSENT

Written informed consent was obtained from the patient for publication of this case report and accompanying images. A copy of the written consent is available for review by the Editor-in-Chief of this journal on request.

## AVAILABILITY OF DATA AND MATERIALS

Available on request.
